# Classifying Promoters by Interpreting the Hidden Information of DNA Sequences via Deep Learning and Combination of Continuous FastText N-Grams

**DOI:** 10.3389/fbioe.2019.00305

**Published:** 2019-11-05

**Authors:** Nguyen Quoc Khanh Le, Edward Kien Yee Yapp, N. Nagasundaram, Hui-Yuan Yeh

**Affiliations:** ^1^Professional Master Program in Artificial Intelligence in Medicine, Taipei Medical University, Taipei, Taiwan; ^2^Singapore Institute of Manufacturing Technology, Innovis, Singapore, Singapore; ^3^Medical Humanities Research Cluster, School of Humanities, Nanyang Technological University, Singapore, Singapore

**Keywords:** DNA promoter, transcription factor, word embedding, convolutional neural network, natural language processing, precision medicine

## Abstract

A promoter is a short region of DNA (100–1,000 bp) where transcription of a gene by RNA polymerase begins. It is typically located directly upstream or at the 5′ end of the transcription initiation site. DNA promoter has been proven to be the primary cause of many human diseases, especially diabetes, cancer, or Huntington's disease. Therefore, classifying promoters has become an interesting problem and it has attracted the attention of a lot of researchers in the bioinformatics field. There were a variety of studies conducted to resolve this problem, however, their performance results still require further improvement. In this study, we will present an innovative approach by interpreting DNA sequences as a combination of continuous FastText N-grams, which are then fed into a deep neural network in order to classify them. Our approach is able to attain a cross-validation accuracy of 85.41 and 73.1% in the two layers, respectively. Our results outperformed the state-of-the-art methods on the same dataset, especially in the second layer (strength classification). Throughout this study, promoter regions could be identified with high accuracy and it provides analysis for further biological research as well as precision medicine. In addition, this study opens new paths for the natural language processing application in omics data in general and DNA sequences in particular.

## Introduction

**Figure 1:**
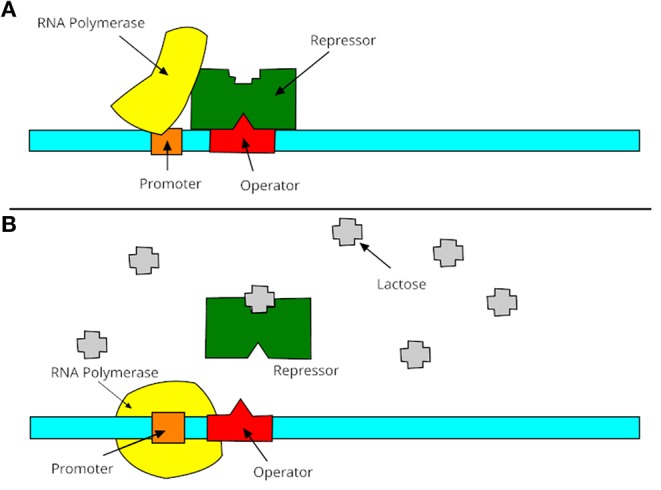
Process of promoters in transcription. **(A)** The gene is essentially turned off. The repressor is not inhibited by lactose and binds to operator, then promoter is bound to make lactase; **(B)** the gene is turned on. The repressor is inhibited by lactose, then the promoter is bound by the RNA polymerase and express the genes to synthesize lactase. Finally, the lactase will digest all of the lactose, until nothing binds to the repressor. The repressor will then bind to the operator, stopping the manufacture of lactase.

A promoter is a region of DNA where RNA polymerase begins to transcribe a gene. Normally, promoter sequences are typically located directly upstream or at the 5′ end of the transcription initiation site (Lin et al., 2018). Both promoters and transcription initiation sites are bound by RNA polymerase and the necessary transcription factors. Promoter sequences describe the direction of transcription and point out which DNA strand will be transcribed (known as sense strand). The transcription process is shown in Figure 1, which contains two steps: turning on and turning off genes. In these two stages, promoters receive information from RNA polymerase to decide the manufacture of lactase. Promoters can be about 100–1,000 base pairs long. There are three elements of promoters in eukaryotic cells, such as core promoter, proximal promoter, and distal promoter. Each of them plays a different role in DNA transcription and RNA polymerase. Many recent studies suggested that DNA promoters may be the primary cause of many human diseases, especially diabetes (Döhr et al., 2005; Ionescu-Tîrgovişte et al., 2015) or Huntington's disease (Coles et al., 1998).

Owing to the huge importance of promoters in genetics and human diseases, the detection of them is an essential problem in genome research. A lot of efforts had been made to address this issue, from researchers with wet-lab, experimental, and computational techniques. One of the most important techniques is to detect the promoters based on TATA box, which is a motif that contains 24% of promoter genes in eukaryotes. Examples of this approach include: Promoter Scan (Prestridge, 1995) built a scoring profile by combining a weighted matrix for scoring a TATA box; Promoter2.0 (Knudsen, 1999) combined genetic algorithms and elements similar to neural networks to recognize promoter regions; Reese (2001) annotated promoters in the *Drosophila* melanogaster genome using a time-delay neural network; and (Down and Hubbard, 2002) combined TATA box with flanking regions of C-G enrichment. Later, some approaches focused on addressing this problem with spatial information of the base pairs in the sequences. There are some examples in this case: PromoterInspector identified promoters, based on the genetic context of promoters rather than their exact location; MCPromoter1.1 (Ohler et al., 1999) identified promoters based on three interpolated Markov chains (IMCs) of a different order. Moreover, the location of GpG islands had been used to predict the promoters region, as shown in Ioshikhes and Zhang (2000), Davuluri et al. (2001), and Ponger (2002).

Over the past decade, with the development of NGS technology, a large number of sequences was transcribed, which motivates researchers to build their predictors on sequence information. Similarly for promoters, it is necessary and urgent to develop highly efficient prediction techniques on it. Some notable research have been reported in the identification of promoters using sequence information. For instance (Li and Lin, 2006) recognized and predicted σ70 promoters in *Escherichia coli* K-12 by using position-correlation scoring matrix (PCSM) algorithm. This problem has been improved upon using variable-window Z-curve composition (Song, 2011) and six local DNA structural properties (Lin et al., 2018). Yang et al. (2017) exploited sex cell types and word embedding to identify enhancer–promoter interaction. Two types of promoters (σ54 and σ28) were identified by integrating DNA duplex stability into neural networks (de Avila e Silva et al., 2014). Later, (Lin et al., 2014) identified σ54 promoters using PseKNC, which is an advanced feature in bioinformatics fields. PseKNC had been used in the latter applications to classify promoter's types (Liu et al., 2017) and promoter's strength (Xiao et al., 2018). The promoter strength of *Escherichia coli* σ70 has been also predicted in Bharanikumar et al. (2018) with use of respective position weight matrices (PWM). Deep convolutional neural networks have been used to identify promoters using sequence information, such as recognition of prokaryotic and eukaryotic promoters (Umarov and Solovyev, 2017).

Identifying promoters, especially their strength, is an important problem in this aspect and latest research (Xiao et al., 2018) has achieved an accuracy of 83.13 and 71.20% for two layers, respectively. However, the performance results are not satisfactory and requires a lot of efforts from bioinformatics researchers to enhance the accuracy. A novel approach, proposed in this study, aims to address this problem. Our idea is based upon the natural language processing (NLP) field which classifies the text/sentence into its appropriate scenario. Therefore, we would like to apply it to bioinformatics to interpret the hidden information of DNA sequences (represented by promoters). Over the past decade, some researchers have successfully applied NLP techniques into biological sequences. One of the pioneering studies is from Asgari and Mofrad (2015) and it had been applied successfully in many later bioinformatics applications (Habibi et al., 2017; Hamid and Friedberg, 2018; Öztürk et al., 2018). However, most studies used the Word2Vec model or FastText model with a single level of N-gram. Here, a novel approach is presented, in which we used a combination of FastText N-grams to represent the DNA sequences. With this idea, we are able to take into account the sub-word information of DNA sequences as well as many N-gram levels in order to aid the increase in the predictive performance. Another point is the use of deep learning to take advantage of the numerous promoter sequences in this problem.

We listed some key contributions of this study which are as follows: (1) a computational model for classifying promoters which achieved better performance than the previous methods; (2) a novel method for generating hidden information of DNA sequences by incorporating a combination of FastText N-grams and deep learning; (3) a study that provides significant information for researchers and biologists to better understand the promoter's functions; and (4) a basis for further study that would apply the FastText model and deep learning architecture in solving the bioinformatics problem. Here we deal with these contributions clearly in the following sections.

## Methods

**Figure 2:**
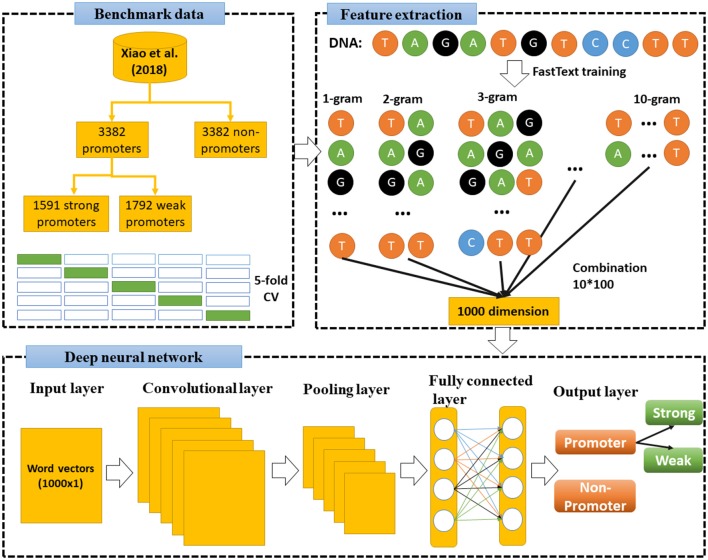
Flowchart of this study. First, we used FastText to train model and extract features from benchmark dataset (Xiao et al., 2018), then combined 10-gram levels to a combination sets of vectors (1,000 dimensions). Deep neural network was then constructed to learn these vectors and classify the DNA sequences.

Under the operation of a specifically designed pipeline, an overall flowchart of our approach is presented in Figure 2. Each of the experimental steps of this proposed pipeline will be sequentially addressed in the following subsections.

### Benchmark Dataset

Collecting a high-quality dataset is one of the most important steps to address a bioinformatics problem. In this study, we re-used the benchmark dataset from Xiao et al. (2018) to objectively assess the difference in performance between our model and other existing ones. In this dataset, they collected all experimentally—confirmed promoter sequences from RegulonDB (Gama-Castro et al., 2015), which is a huge database of the regulatory network of gene expression. These sequences were categorized into two groups: strong and weak promoters based on their levels in transcription activation and expression. They also extracted non-promoter sequences by considering intron, exon, and intergenic sequences excluding the positive sequences. After that, the CD-HIT [26] was also used to exclude the pairwise sequences whose similarities were calculated to be more than 85%.

The benchmark dataset encompasses 3,382 promoter samples and 3,382 non-promoter samples. In 3,382 promoter samples, there are 1,591 strong promoter samples and 1,792 weak promoter samples for construction of second layer classification. It can be freely downloaded at http://www.jci-bioinfo.cn/iPSW(2L)-PseKNC/images/Supp.pdf. The whole dataset was randomly divided into five subsets to perform a 5-fold cross-validation. The training process was performed using a fixed ratio of the training set over the validation set of 4:1 with alternation.

### DNA Representation With Language Model

A DNA sequence consists of four nucleotides: adenosine (A), cytidine (C), guanosine (G), and thymine (T). These nucleotides will combine together to form a definite sequence in the DNA sequence. Feature extraction is an important step in most of the bioinformatics problems, whereby the main features will help in discriminating DNA sequences. One of the most common methods is the use of k-mer. K-mers are described as all the possible subsequences (of length k) from a read accessed through DNA sequencing. The number of k-mers possible given a string of length L is L-k+1, whilst the number of possible k-mers given n possibilities (four in the case of DNA e.g., ATGC) is nk. K-mer has been used in a lot of bioinformatics problems and has achieved promising results. Next, Chou highlighted PseDNC which has extracted DNA sequences via different ways. PseDNC has helped to rectify numerous problems relating to bioinformatics, as compared to using k-mer. Another approach is the use of language model to represent the information of DNA sequences. In this approach, DNA sequence will be treated as a language sentence and then fed into supervised learning for classification. We can easily list the methods using this approach, from Word2Vector to FastText. In these approaches, FastText has been proven to achieve better performance as compared to Word2Vector or Glove.

### FastText Implementation

In order to generate continuous N-grams, we made use of FastText (Bojanowski et al., 2017), which is a library from Facebook for representation and classification of text. In FastText, we can train different language models such as skip-gram or CBOW and apply a variety of parameters such as sampling or loss functions. There are a lot of improvements from Word2Vector to FastText as described in Bojanowski et al. (2017) and Le et al. (2019a). In this study, each DNA sequence was treated as a sentence with a lot of words. Moreover, each word contains a bag of character n-gram. As mentioned in FastText's document, they modified the algorithm of Word2Vector whereby special symbols “and” are added at the boundary of words, which helps to differentiate prefixes and suffixes from other character sequences. Moreover, the word itself has been also included in the n-gram set to learn a representation for each word (together with character n-grams). To explain the idea, we used our DNA word “ATGAC” as an example. If we would like to generate the representation of this word with 3-gram, they will be consequently: <AT, ATG, TGA, GAC, AC> and the special sequence <ATGAC>. Here, it is noteworthy that the representation <TGA>, corresponding to the word “TGA,” is different from the tri-gram “TGA,” derived from the word “ATGAC.” The reason is because of the potential of extracting sub-word information in word “TGA” of FastText and it could help generate more information for each word. The word generated by FastText could be considered as a continuous bag of words. In this study, we extracted all the n-grams from 1 to 10 to consider the optimal levels of them.

What makes FastText different from Word2Vector is the sub-word information, and it is proposed via a scoring function *s* as follows:

(1) $$\begin{array}{r} s\left(w,c\right)=\underset{g\in G_{w}}{\sum }z_{g}^{T}v_{c} \end{array}$$

where G is the size of n-grams, G_w_ ranges from 1 to G, w is a given word, z_g_ is a vector representation to each n-gram g, v_c_ is context vector. This simple modification allows objective representation of words, thus helping the model learn reliable representation for rare words.

Based on the recent successful applications of FastText model in representing biological sequence (Le, 2019; Le et al., 2019a), we introduced a more in-depth benchmark method using FastText to improve this representation. Here we take into account the combination of continuous N-gram levels, which was not considered by the previous studies. It means that instead of using only one level of N-gram and sub-word information, we used a lot of N-gram combinations and considered which was the best combination for this problem. A huge advantage of this approach is that we can have many features for learning. In addition, we can easily implement feature selection techniques and improve the performance results in the model.

### 1D Convolutional Neural Network

In general, CNN is a class of deep neural networks that has been demonstrated to be exceptionally successful in territories, such as picture acknowledgment and order. CNN has been fruitful in computer vision related issues such as face recognition, object detection, or self-driving cars. CNN appears ready to reproduce and upgrade these key strides in a bound together structure and learn various leveled portrayals specifically from crude images. If we take a convolutional neural organization that has been prepared to perceive protests inside pictures, then that system will have built up some inward autonomous portrayals of the substance and style contained inside a given picture. Since the input of this problem was a vector, therefore, we used 1D CNN. Similar to 2D CNN approaches which has been used in bioinformatics (Le and Nguyen, 2019; Le et al., 2019b; Nguyen et al., 2019), it consisted of the following layers:

1. Input layer: The input of our model is a 1D vector, which is a vector of size 1 × 100 (created by FastText model).
2. Convolutional layer: A 1D convolutional layer (e.g., temporal convolution) is used to construct a convolution kernel and then derive features encoded in the 1D input vector. The convolutional layer moves in stride over the input, transforming the values into representative values via a sliding window. This process helps conserve the dimensional relationship between numeric values in the vectors, by gaining beneficial features using small parts of input data. Since our input size was not big, a kernel size of 3 was applied to figure out more information.
3. Rectified Linear Unit (ReLU): an additional non-linear operation is presented after every convolution operation. It aims to perform non-linear function in our CNN and help our model understand data better. The output function of ReLU is as follows:
  (2) $$\begin{array}{r} f\left(x\right)=\max\left(0,x\right) \end{array}$$
  where x is the number of inputs in a neural network.
4. Pooling layer: It is normally added inside the convolutional layers to reduce the calculation of the next layers. Max pooling was selected in this step with stride of 2.
5. Dropout layer: A technique which aims to prevent overfitting and also help to increase the model's performance (Srivastava et al., 2014).
6. Flatten layer: a layer helps to transform the input matrix into a vector.
7. Fully connected layer: is normally inserted by the last stage of the deep networks. The layer is fully-connected if each node is connected with all of the previous nodes in the network. Our problem is to identify between promoter and non-promoter (or classify strong and weak promoter), thus it was a binary classification. Therefore, the final number of nodes in our output is 2.
8. Softmax is a logistic function defined by the formula:
  (3) $$\begin{array}{r} {\sigma \left(z\right)}_{i}=\frac{e^{z_{i}}}{\sum_{k=1}^{K}e^{z_{k}}} \end{array}$$
  where z is the input vector with K-dimensional vector, σ(z)_*i*_ is real values in the range (0, 1) and ith class is the predicted probability from sample vector x. It was compulsory to insert Softmax, in order to determine the probability of each possible output.

### Assessment of Predictive Ability

To evaluate the performance of the classifiers that were constructed by the aforementioned deep learning architecture, the 5-fold cross-validation technique was implemented. The average metrics among the five testing sets were determined in order to compare the performance when constructing the classifier. We follow Chou's evaluation criteria which is widely used in many bioinformatics studies (Chou, 2001; Xiao et al., 2018; Le et al., 2019a). The criteria includes sensitivity (Sens), specificity (Spec), accuracy (Acc), and Matthews Correlation Coefficient (MCC) which are defined as:

(4) $$\begin{array}{r} Sensitivity=1-\frac{N_{-}^{+}}{N^{+}}\;,\;0\leq Sen\leq 1 \end{array}$$

(5) $$\begin{array}{r} Specificity=1-\frac{N_{+}^{-}}{N^{-}}\;,\;0\leq Spec\leq 1 \end{array}$$

(6) $$\begin{array}{r} Accuracy=1-\frac{N_{-}^{+}+N_{+}^{-}}{N^{+}+N^{-}}\;,\;0\leq Acc\leq 1 \end{array}$$

(7) $$\begin{array}{r} MCC=\;\frac{1-\left(\frac{N_{-}^{+}}{N^{+}}+\frac{N_{+}^{-}}{N^{-}}\right)}{\sqrt{\left(1+\frac{N_{+}^{-}-N_{-}^{+}}{N^{+}}\right)\left(1+\frac{N_{-}^{+}-N_{+}^{-}}{N^{-}}\right)}}\;,\;-1\leq MCC\leq 1 \end{array}$$

The relations between these symbols and the symbols in Equations (4, 5, 6, and 7) are given by:

(8) $$\{\begin{array}{c} N_{+}^{-}=FP\\ N_{-}^{+}=FN\\ N^{+}=TP+N_{-}^{+}\\ N^{-}=TN+N_{+}^{-} \end{array}$$

True positive (TP) and true negative (TN) are the respective numbers of correctly predicted promoter and non-promoter, whereas false positive (FP) and false negative (FN) are the respective numbers of misclassified promoter and non-promoter.

Likewise, we also used Receiver Operating Characteristics (ROC) curve and Area Under Curve (AUC) (Bradley, 1997) as the additional metrics for performance evaluation. The AUC is a probability value ranging from 0 to 1 in which the greater AUC shows the better predictive performance.

## Results

### Optimal Experimental Setup

**Figure 3:**
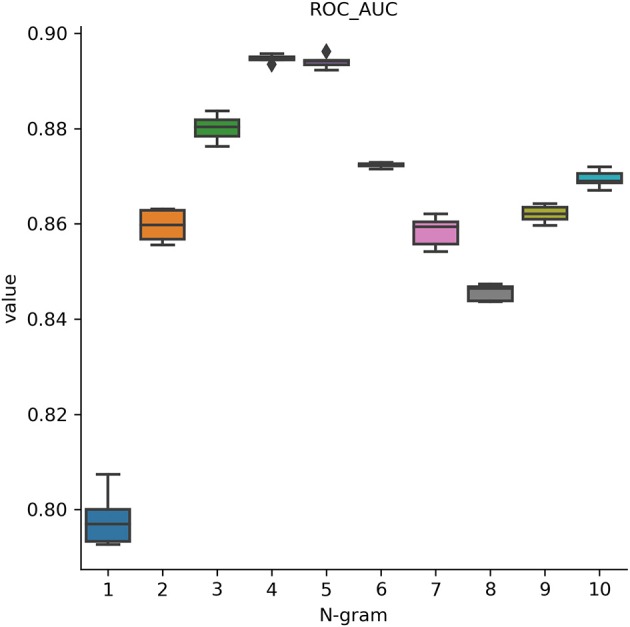
Performance results on identifying promoters using different levels of N-gram. Our classifier could classify promoters with high performance (AUC ~ 0.9), especially at 4-gram and 5-gram levels.

In this analysis, we attempted to observe the optimal hyperparameters that were used in this study. Because we integrated FastText and deep learning model, we chose the best parameters for both methods. FastText has a lot of different parameters for training purpose. Many prior research on it determined that changing these parameters will help to change the model's accuracy drastically. Therefore, we would like to perform a one-by-one strategy to tune up the optimal parameters in FastText. There are a lot of parameters that may affect the performance results and we decided to adapt these parameters such as wordNgrams (max length of word n-gram), lr (learning rate), dim (size of word vectors), ws (size of context window), epoch (number of iterations), and loss (loss function). We used a basic setting on FastText classifier to perform supervised learning for text classification. The dataset used in this section helped distinguish between promoters and non-promoters. In the first experiment, we would like to examine the effect of different levels of N-grams (from 1 to 10) on the performance results. The important measurement metric used in this evaluation is ROC AUC value. As shown in Figure 3, our classifier could classify promoters with high performance (AUC ~ 0.9), especially in two levels: 4-gram and 5-gram. However, the differences were not significant and it indicates that we can select any level of N-gram to create a good model for promoter classification. Table 1 shows the hyperparameters used for tuning the model. After the tuning process, we also presented the best set of hyperparameters found: learning rate of 0.1, vector dimension of 100, context window size of 5, epoch of 100, and softmax loss function.

**Table 1:** Hyperparameters chosen for tuning FastText model.

**Parameters**	**Range**	**Stepsize**	**Optimal**
lr	0.05–0.25	0.05	0.1
Dim	50–500	25	100
Ws	1–10	1	5
Epoch	25–500	25	100
Loss	[ns, hs, softmax]	-	softmax

*Lr, learning rate; dim, dimension; ws, size of context window; epoch, number of iterations; loss, loss function*.

The next tuning is from deep learning architecture, in which we performed a grid search CV on a set of potential hyperparameters. All of the parameters selected for tuning in CNN include the number of layers, epochs, batch sizes, dropout values, weight constant as well as the optimizer and activation function. After this step, we identified a set of optimal hyperparameters in CNN as follows: 64 filter layers, batch size of 100, epoch of 100, dropout of 0.3, weight constraint of 4, adadelta optimizer, and linear activation. We then used all of the optimal parameters in the next experiments as well as the later comparisons.

### Effects of Different Levels of N-Gram and Combination of Continuous N-Grams in Classifying Promoters

According to the previous section, changing the number of N-grams did not make significant effect on promoter classification. It has been also proven in some of the previous works which used the FastText model (Le, 2019; Le et al., 2019a). However, one novel idea implemented in this study was to increase the performance results by using a combination of N-grams. The idea was to combine all of the N-gram levels into a big set of features, which will then be fed into classifiers. As such, our classifier will take full advantage of important features for each specific N-gram level and remove some less important features inside all of the levels. The performance results were shown in detail in Table 2. It is noted that the 5-fold cross-validation has been performed for several independent iterations to give a confidence interval for the results. In these results, we fed all 1,000 features from 10 levels of N-gram into our CNN architecture. It is easy to say that the combination of N-grams outperforms the single level of N-gram. This method achieved a sensitivity of 82.76%, specificity of 88.05%, accuracy of 85.41% and MCC of 0.709, which is improved ~1–4% from single N-gram in term of specificity, accuracy, and MCC. To statistically compare between N-gram combination and N-gram single levels, we performed 10 times of one-sided Wilcoxon tests of the ROC AUC values between the combination model and each of the 1–10-gram model. After that, all of Wilcoxon tests showed a *p*-value of 0.0005 (less than significance level ∝ = 0.05) which could strongly conclude that the performance results of combination features were significantly better than the single ones at high confidence level.

**Table 2:** Comparison between single N-gram and combination of continuous N-grams.

**Methods**	**Sens**	**Spec**	**Acc**	**MCC**
Single N-gram	82.43	83.34	82.88	0.658
Combination of N-grams	82.76	88.05	85.41	0.709

*Single N-gram, representative by 4-gram; Combination of N-grams, combine 10 levels of N-gram together*.

Since deep learning is a black-box manner, it automatically generated the hidden information from our feature sets. Therefore, it is challenging to understand which features have most contribution or play critical role for promoter distinction in our model. As a reference, we used a common technique namely Maximum-Relevance-Maximum-Distance (MRMD) (Zou et al., 2016a) to evaluate and extract the important features of our datasets. MRMD has been used a lot of works in bioinformatics with promising results (Zou et al., 2016b; Wei et al., 2017). According to the results, MRMD suggested that our model will reach the highest accuracy when we selected 835 top-ranked features (out of 1,000) to insert into our neural network. To detail, 10 features had the highest scores were shown in Table 3. These features, therefore, play an essential role in classifying promoter sequences using our model.

**Table 3:** Top-ranked features using MRMD feature selection technique.

**No**.	**Feature number**	**Score**
1	feature_97	1.0
2	feature_21	0.9170726107858075
3	feature_34	0.9096134637807235
4	feature_92	0.8914645287023287
5	feature_54	0.8463944338892277
6	feature_9	0.8368290059895386
7	feature_41	0.824726606348234
8	feature_8	0.8020998165541897
9	feature_77	0.7714372077391476
10	feature_3	0.7598084153408637

Next, we would like to compare our performance results with a baseline machine learning technique to check whether the deep CNN has generated more hidden information and given a significant performance. Since nearest neighbor (kNN) (Keller et al., 1985) has been used to represent for traditional machine learning classifiers in different problems, we implemented it in our study for comparison. We used hyperparameter optimization process and found that the model performed consistently at 10 neighbor trees. The optimal performance reached 78.8%, 86.8%, 82.8%, 0.66, and 0.885 for sensitivity, specificity, accuracy, MCC, and AUC, respectively. Compared with the performance from CNN, kNN was lower in term of sensitivity, accuracy, MCC, and AUC. It is enough evidence to say that the deep neural network could learn more features and produce a better performance than traditional neural networks.

### Classifying Promoters' Strength

Since the combination of N-grams performed well in the first layer classification, we aimed to use the same experimental setups for the second layer (classifying promoter's strength). Our dataset includes 1591 strong promoters and 1792 weak promoters as collected from Xiao et al. (2018) and has been mentioned in the dataset section. The experiments show that our method, which used a combination of N-grams, could classify the promoter's strength with an accuracy of 73.1%, sensitivity of 69.4%, specificity of 76.4%, and MCC of 0.46. The performance was also better than the baseline models with single levels of N-grams. It means that we can use this setup for both layers with promising results.

### Comparison the Performance Results Between Proposed Method and the Existing Methods

Our best model as mentioned in the previous sections is the combination of different N-gram levels and deep convolutional neural networks. To be fair, we have to compare our proposed method with the other previous works that regarding promoter classification. Also it is noted that we surely chose the previous works that used the same benchmark dataset. For the first layer, numerous studies had been done, including PCSF (Li and Lin, 2006), vw Z-curve (Song, 2011), Stability (de Avila e Silva et al., 2014), iPro54 (Lin et al., 2014), iPromoter-2L (Liu et al., 2017), and iPSW(2L)-PseKNC (Xiao et al., 2018). Among these studies, only the last one performed the classification of promoter's strength, thus we also compared with this predictor in our second layer. The results are shown in Table 4, and we highlighted the highest values to highlight the significance of each metrics. We then observed that our method outperforms other predictors in all metrics (sensitivity, specificity, accuracy, and MCC) in both layer classifications. Another improvement is that our approach could be applied to actual genome sequences (long fragments of bacterial genomes) rather only short sequences. All sequences with different length will be trained to become a vector with a fix-length. It helps to input any form of sequences flexibly.

**Table 4:** Comparison with previous predictors on the same benchmark dataset.

**Predictors**	**Sens**	**Spec**	**Acc**	**MCC**
**1st layer**				
Ours	**82.76**	**88.05**	**85.41**	**0.709**
iPSW(2L)-PseKNC	81.37	84.89	83.13	0.663
iPromoter-2L	79.2	84.16	81.68	0.6343
iPro54	77.76	83.15	80.45	0.61
Stability	76.61	79.48	78.04	0.5615
vw Z-curve	77.76	82.8	80.28	0.6098
PCSF	78.92	70.7	74.81	0.498
**2nd layer**				
Ours	**69.4**	76.4	**73.1**	**0.46**
iPSW(2L)-PseKNC	62.23	**79.17**	71.2	0.4213

Highlighted values are the significant values for each metric.

## Discussions

Promoters play an important role in the transcription of genes affect numerous human diseases. Therefore, identification of promoters using their sequence information is one of the most important tasks in bioinformatics. Although few computational tools had already been presented, the performance results require improvements. This study presents a new hybrid system, from deep learning and a combination of FastText N-grams, to identify promoters and their respective strengths. To our knowledge, this is the first bioinformatics study which has applied this hybrid into biological sequences. By using this method, we are able to generate the hidden information of DNA sequences unlike other methods. Our performance results were evaluated via a 5-fold cross-validation test on a benchmark dataset. It was found that the proposed method could identify promoters and their strength, with an accuracy of 85.41 and 73.1%, respectively. The rest of the measurement metrics, such as sensitivity, specificity, and MCC, also attained superior performances. When compared to the other state-of-the-art predictors regarding the same problem and dataset, our proposed method has improved at about 1–4% in all of the metrics. Therefore, our model can be considered as a reliable method for identifying promoters and their strength, with use of sequence information. It can also act a basis for further study that aims to interpret the language context of DNA sequences.

Last but not least, scientists can use our approach to solve further bioinformatics problems on sequencing. Since most bioinformatics problems focused on sequencing data, their features could be extracted by using our combination (different levels of FastText N-grams). They then be fed into a supervised learning to perform the prediction or classification (e.g., using deep neural network as proposed in this work). It could also provide a new approach for the previous works that only used one level of FastText (Le, 2019; Le et al., 2019a). A combination of more levels could be a solution for boosting their predictive performances. We also provided our source codes at https://github.com/khanhlee/deepPromoter to help reproducing our method. Furthermore, since a lot of previous works on promoter classification extracted features by using PseKNC [such as (Liu et al., 2017; Lin et al., 2018; Xiao et al., 2018)], a hybrid of this feature and our features could be considered in the future works for the purpose of performance improvement.

## Data Availability Statement

Publicly available datasets were analyzed in this study. This data can be found here: http://www.jci-bioinfo.cn/iPSW(2L)-PseKNC/images/Supp.pdf.

## Author Contributions

NL and EY conceived the ideas and designed study. NL conducted the experiments and analyzed the results. NL, EY, NN, and H-YY participated in the discussion of the results and writing of the article. All authors read and approved the final version of the manuscript.

### Conflict of Interest

The authors declare that the research was conducted in the absence of any commercial or financial relationships that could be construed as a potential conflict of interest.
